# The Southern Branch of the National Dental Association

**Published:** 1898-04

**Authors:** 


### The Southern Branch of the National Dental Association.

The Southern Branch of the National Dental Association held its first annual meeting in St. Augustine, Fla., February 22d to 25th. A revised constitution was adopted harmonizing in all its features (including the delegate system of membership) with that of the National Association.

The following officers were elected to serve the ensuing year: President, Dr. Wm. E. Walker, Pass Christian, Miss.; First Vice-President, Dr. T. P. Hinman, Atlanta, Ga.; Second Vice-President, Dr. H. H. Johnson, Macon, Ga.; Third Vice-President, Dr. E. F. Adair, Harmony Grove, Ga.; Treasurer, Dr. B. D. Brabson, Knoxville, Tenn.; Corresponding Secretary, Dr. C. L. Alexander, Charlotte, N. C.; Recording Secretary, Dr. S. W. Foster, Atlanta, Ga.; Executive Committee, (the two new members to serve three years), Dr. I. Simpson, Rock Hill, S. C. and Dr. E. G. Quattlebaum, Columbia, S. C.

Time and place of next meeting, New Orleans, La., February 15th (the day after Mardi Gras).

